# Supplementary material for: Improved Multimodal Tumor Necrosis Imaging with IRDye800CW-DOTA Conjugated to an Albumin-Binding Domain
Source: Cancers (Basel). 2022 Feb 9;14(4):861. doi: 10.3390/cancers14040861 (PMC8870237; doi:10.3390/cancers14040861)
Supplement: Supplementary file 1 [file cancers-14-00861-s001.zip › cancers-1526162-supplementary.pdf]

Supplementary data

1. Synthesis

1.1. General information

HPLC analyses were performed on a system from Waters (Milford, MA, USA) equipped with a quaternary pump, a 2998 PDA detector, a radio detector consisting of a NaI crystal detector with a Canberra Osprey-DTB, and dedicated software. The following HPLC methods have been used:

- HPLC method A is Symmetry C_{18} analytical column (4.6 × 250 mm, 5 μm, Waters) and a gradient profile of 0–1 min 10% B, 1–20 min towards 60% B at a flow rate of 1.0 mL/min. Solvent A was water + 0.1% TFA (v/v), and solvent B was ACN + 0.1% TFA (v/v) for method A, B and C.
- HPLC method B is Gemini C_{18} semi-preparative column (10 × 250 mm, 5 μm, Waters) and a gradient profile of 0–1 min 20% B in A, 1–25 min to 30% B at a flow rate of 3.0 mL/min.
- HPLC method C Gemini C_{18} semi-preparative column (10 × 250 mm, 5 μm, Phenomenex) and a gradient profile of 0–1 min 25% B in A, 1–25 min to 40% B at a flow rate of 3.0 mL/min .
- HPLC method D is Symmetry C_{18} analytical column (4.6 × 250 mm, 5 μm, Waters) and a gradient profile of 0–1 min 42% B, 1–20 min towards 61% B at a flow rate of 1.0 mL/min. Solvent A was 0.2 M Tris-HCl buffer pH 8.5 + 10% MeOH and solvent B was MeOH.

ESI-MS analyses were performed on a TSQ Quantum Ultra system equipped with a Surveyor Autosampler Plus and MS Pump Plus from Thermo Fisher Scientific (San Jose, CA, USA).
Chemical names were generated by Chemdraw (version 17.1.1.0)

1.2. 2,2',2''-(10-(2-(((*S*)-1-(((*S*)-1,6-diamino-1-oxohexan-2-yl)amino)-6-(4-(4-iodophenyl)butanamido)-1-oxohexan-2-yl)amino)-2-oxoethyl)-1,4,7,10-tetraazacyclododecane-1,4,7-triyl)triacetic acid (4, Scheme 1, steps a-h)

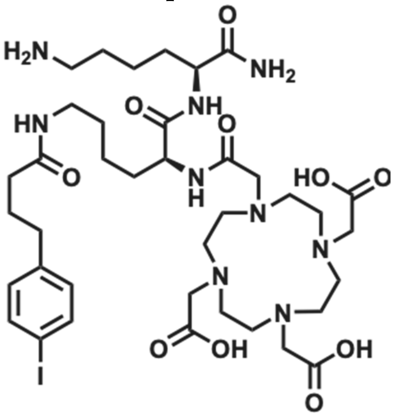

The title compound was synthesized beginning by the deprotection of the Rink amide MBHA resin (222 mg, loading capacity 0.65 mmol/g) in 20% piperidine in DMF (1 x 10 min., 1 x 50 min). After washing thrice with DMF, Fmoc-L-Lys(Boc)-OH (68 mg, 0.15 mmol) was loaded on the solid support in the presence of HBTU (220 mg, 0.58 mmol) and DIPEA (150 µL, 1.16 mmol). The reaction mixture was left to stir for 3 h at room temperature. The remaining amine groups were capped with a mixture of acetic anhydride (686 µL, 7.26 mmol) and DIPEA (3.7 mL, 21.8 mmol) for 15 min. Fmoc-protecting group was removed by two subsequent treatments (1 × 10 min., 1 × 50 min) of the resin with 20% piperidine in DMF (5 mL), followed by three washes with DMF. Then, ivDde-L-Lys(Fmoc)-OH (250 mg, 0.58 mmol) was coupled to the peptidyl resin in the presence of OximaPure (82.5 mg, 0.58 mmol), HBTU (220 mg, 0.58 mmol), and DIPEA (202 µL, 1.16 mmol) in DMF (1.5 mL). The reaction was monitored until the Kaiser test was found negative. Fmoc-protecting group was removed with 20% piperidine in DMF (10 mL), as previously described. Subsequently, 4-(*p*-iodophenyl)butyric acid (168.4 mg, 0.58 mmol) was coupled to the deprotected lysine-residue in the presence of OximaPure (82.5 mg, 0.58 mmol), HBTU (220 mg, 0.58 mmol) and DIPEA (202 µL, 1.16 mmol) in DMF (1.5 mL). Completion of the reaction was monitored by Kaiser test. The ivDde-protecting group was removed by addition of a 2% solution of hydrazine in DMF (5 mL), and the reaction mixture was stirred for 10 min at rt. Then, the reaction vessel was emptied, filled again with the hydrazine solution, and stirred for 2 hours. The resulting free amine was then coupled to DOTA-tris(*t*Bu)ester (CheMatech, Dijon, France, 333 mg, 0.58 mmol) in the presence of OximaPure (82.5 mg, 0.58 mmol), HBTU (220 mg, 0.58 mmol), and DIPEA (202 µL, 1.16 mmol) in DMF (1.5 mL). Reaction progress was monitored by Kaiser test. The peptide was cleaved from the resin and deprotected by the addition of TFA:iPr$_3$SiH:H_2O (95:2.5:2.5, v/v/v), and the formation of product was monitored by ESI-MS. When full deprotection occurred, the filtrate was collected and the resin was rinsed with additional cleavage cocktail. The combined fractions were concentrated under vacuum and purified by semi-preparative HPLC. Product was obtained as a white solid (6.8 mg, 7.3 µmol, 4.9%). HPLC (method A): t_R 11.6 min. ESI-MS: m/z calculated for $C_{38}H_{62}IN_9O_{10}$ was 931.37, m/z found was 953.92 $[M+Na]^+$, 932.08 $[M+H]^+$, 477.58 $[M+H+Na]^{2+}$, 466.31 $[M+2H]^{2+}$.

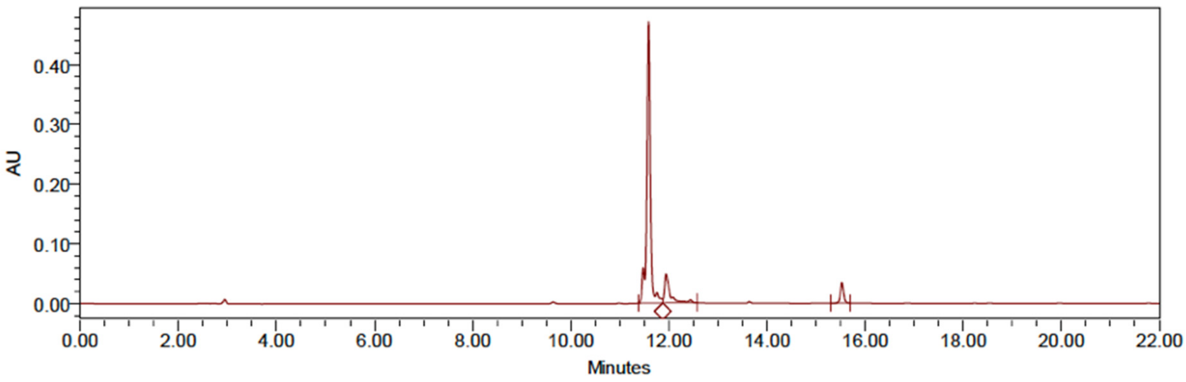

Figure S1: HPLC chromatogram of intermediate compound **4**.

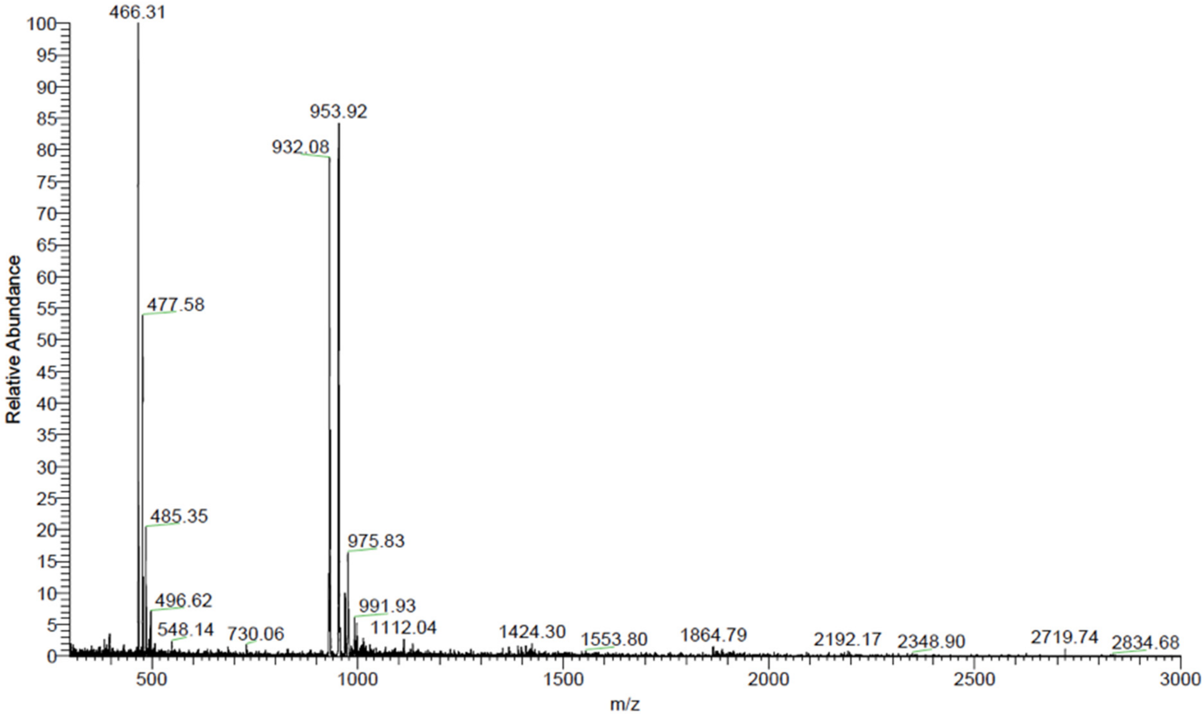

Figure S2: ESI-MS analysis of compound **4**.

1.3. DOTA-L-Lys(4-(*p*-iodophenyl)butyrate)-L-Lys(IRDye800CW)-NH_2 (2, Scheme 1, step i)

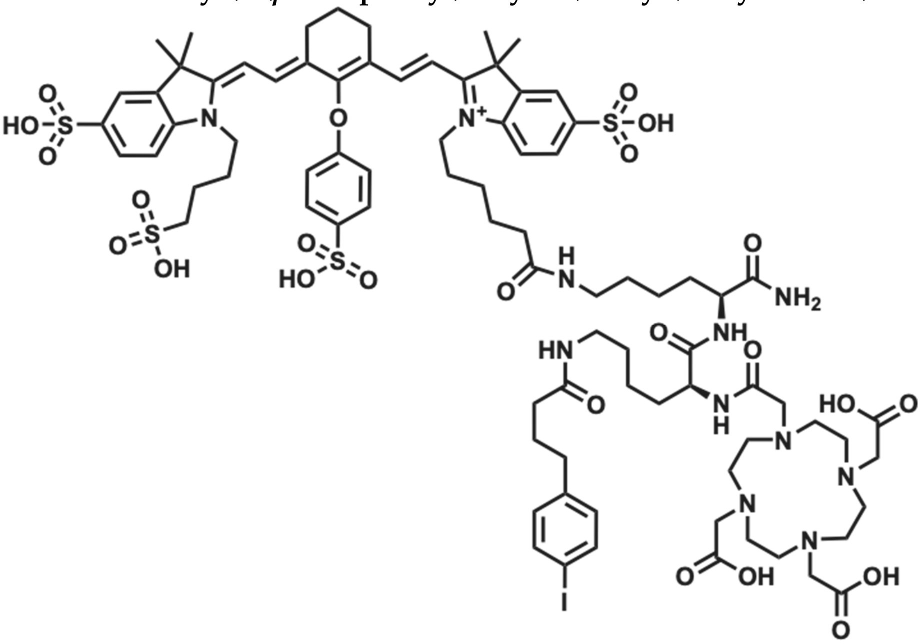

Compound **4** (0.95 mg, 1.02 µmol) and DIPEA (0.4 µL, 2.4 µmol) were dissolved in DMSO (50 µL) and added to IRDye800CW-NHS ester (1.02 mg, 0.93 µmol). The resulting mixture was stirred overnight at room temperature and directly purified by semi-preparative HPLC (Method B, t_R 18.9 min). Product was obtained as a green solid (1.26 mg, 657 nmol, 64%). The precursor was titrated and stored in 10 nmol aliquots at -20 °C. Purity was determined by HPLC (method A): purity >99% (t_R 13.1 min). ESI-MS: m/z calculated for $C_{84}H_{115}IN_{11}O_{24}S_4^+$ was 1916.60, m/z found was 978.35 $[M+H+K]^{2+}$, 969.95 $[M+H+Na]^{2+}$, 958.54 $[M+2H]^{2+}$.

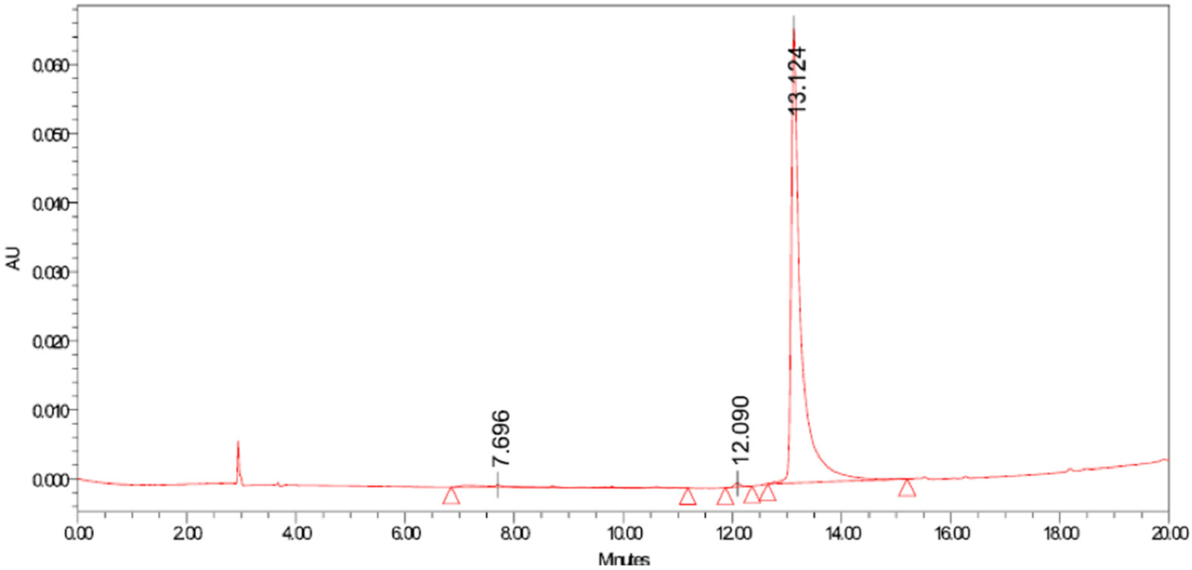

Figure S3: HPLC chromatogram of precursor **2**.

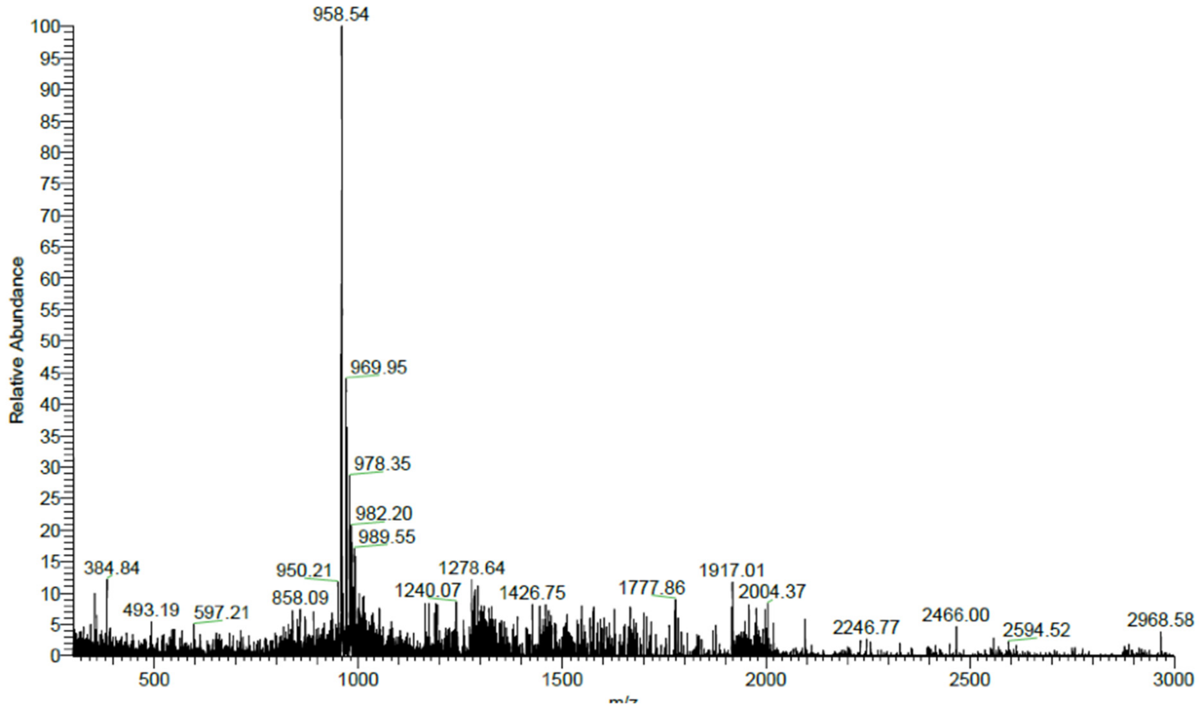

Figure S4: ESI-MS analysis of precursor **2**.

1.4. *N*6-(*tert*-butoxycarbonyl)-*N*2-(4-(4-iodophenyl)butanoyl)-*L*-lysine (5, Scheme 2, steps a-d)

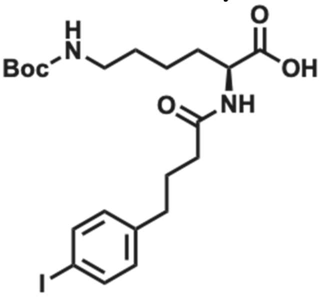

Compound **5** was synthesized by loading Fmoc-L-Lys(Boc)-OH (937 mg, 2.0 mmol) onto 2-chlorotrityl resin (1.0 g loading capacity 0.67 mmol/g) in presence of DIPEA (480 µL, 5 mmol) and DCM (10 mL). The reaction mixture was stirred overnight at room temperature (Scheme 2, a). The remaining chloride groups were capped with MeOH:DIPEA:DCM (15:5:80, v:v:v, 10 mL) for 15 min. Fmoc-groups were removed with 20% piperidine in DMF (10 mL, Scheme 2, b). Then, 4-(*p*-iodophenyl)butyric acid (1.16 g, 4.0 mmol) was conjugated to the primary amine in DMF (10 mL) in the presence of OximaPure (568 mg, 4.0 mmol), HBTU (917 mg, 3.9 mmol), and DIPEA (767 µL, 8.0 mmol) (Scheme 2, c). The protected peptide was cleaved from the resin by the addition of hexafluoroisopropanol (HFIP) in DCM (1:1, v:v, 10 mL) for 1 h and the reaction mixture was filtered (Scheme 2, d). The resin was rinsed with additional HFIP–DCM (2 × 10 mL) and the combined fractions concentrated under vacuum to yield a white solid (828 mg, 1.60 mmol, 80%). HPLC (method A): purity = 98%, t_R 19.0 min. ESI-MS: m/z calculated for $C_{21}H_{31}IN_2O_5$ was 518.14 m/z found was 517.06 $[M-H]^-$. ^{1}H NMR (400 MHz, Chloroform-*d*) δ 7.58 (d, *J* = 8.2 Hz, 2H), 6.92 (d, *J* = 8.2 Hz, 2H), 4.72 – 4.49 (m, 2H), 3.10 (s, 2H), 2.58 (t, *J*= 7.4 Hz, 2H), 2.24 (s, 2H), 1.97 – 1.71 (m, 4H), 1.52 – 1.46 (m, 2H), 1.43 (s, 9H), 1.40 – 1.34 (m, 2H).

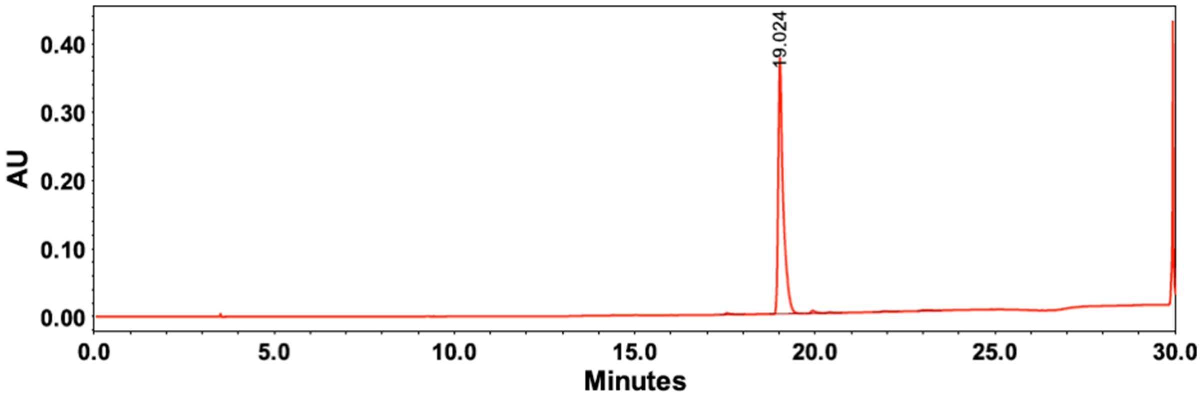

Figure S5: HPLC chromatogram of intermediate compound **5**.

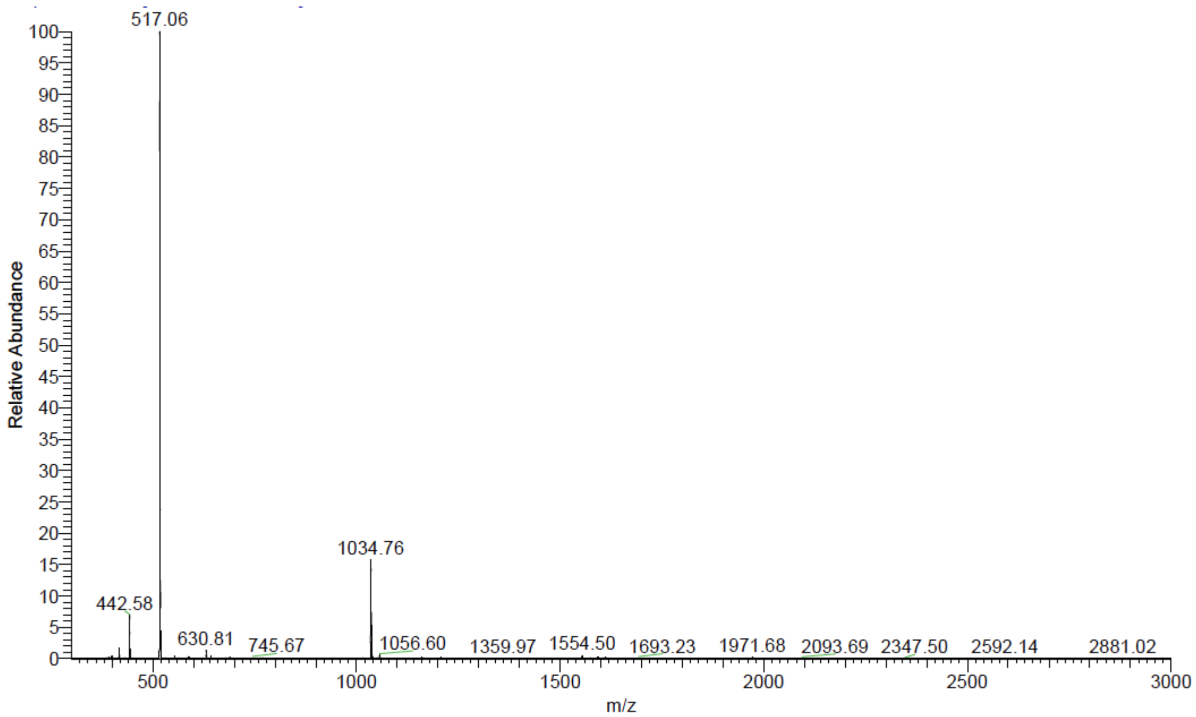

Figure S6: ESI-MS analysis of compound **5**.

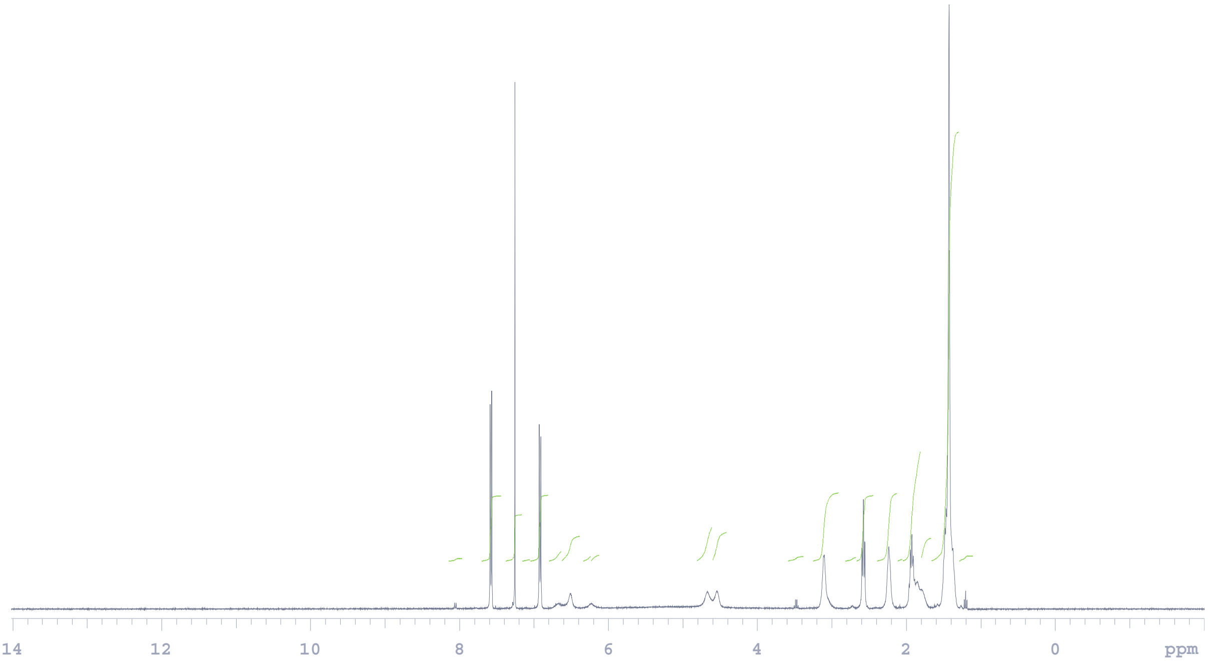

Figure S7: 1H-NMR spectrum compound **5**.

1.5. *tert*-butyl(*S*)-(6-((4-(aminomethyl)benzyl)amino)-5-(4-(4-iodophenyl)butanamido)-6-oxohexyl)carbamate (6, Scheme 2, step e)

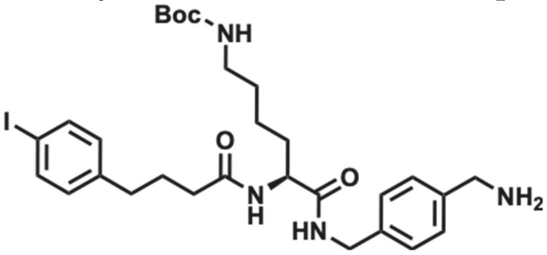

Compound **6** was synthesized by dissolving compound **5** (200 mg, 386 µmol) in DMF (25 mL). EDCi (120 mg, 772 µmol), NHS (88.8 mg, 772 µmol), and DIPEA (135 µL, 970 µmol) were added and the resulting mixture was stirred at room temperature for 2 hours. Then, the mixture was concentrated under reduced pressure, diluted with brine and extracted thrice with EtOAc. The combined organic layers were dried over anhydrous $MgSO_4$, filtered, concentrated under reduced pressure, and dissolved in DMF (5 mL). This mixture was added dropwise to a solution of *p*-xylylene diamine (537 mg, 3.86 mmol) in DMF (10 mL) at 0 ˚C. The resulting mixture was allowed to warm to room temperature overnight. The DMF was removed under reduced pressure and the crude material was then extracted with EtOAc and washed with brine. The organic layer was dried over anhydrous $MgSO_4$, filtered, and concentrated under reduced pressure to obtain a white solid (217 mg, 340 µmol, 88%). The material was used as obtained in the following step. HPLC (method A): t_R 15.6 min. ESI-MS: m/z calculated for $C_{29}H_{41}IN_4O_4$ was 636.22, m/z found was 636.69 $[M+H]^+$.

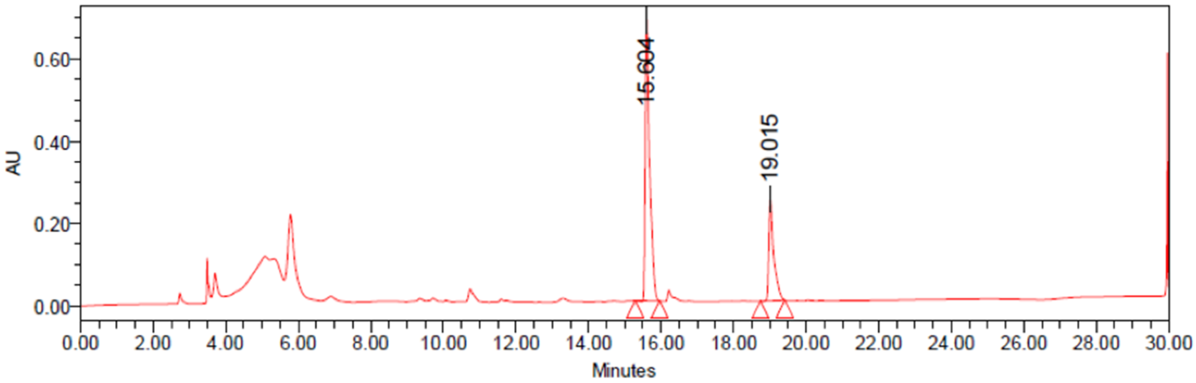

Figure S8: HPLC chromatogram of intermediate compound **6**.

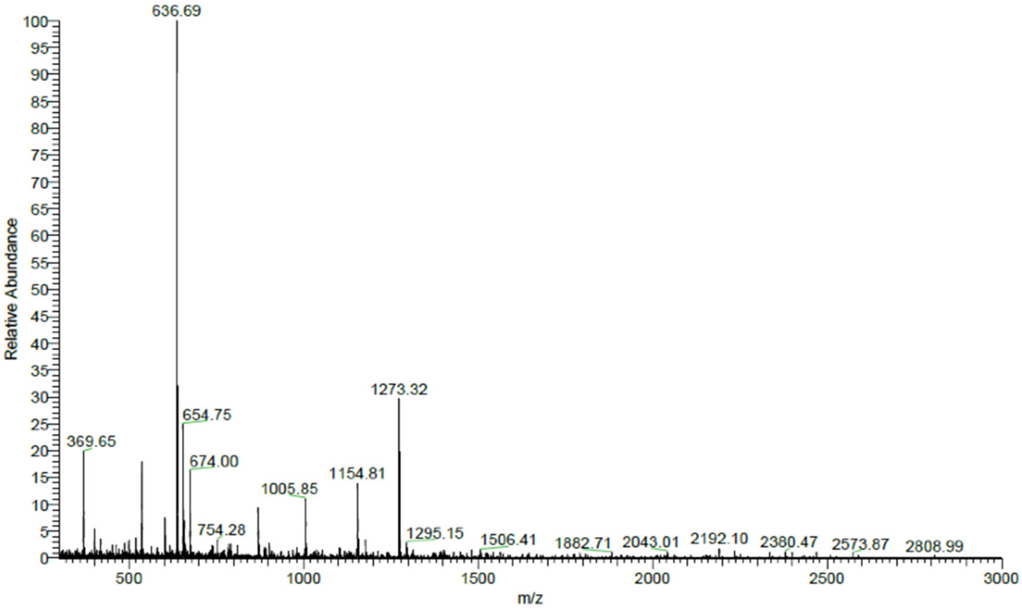

Figure S9: ESI-MS analysis of compound **6**.

1.6. tri-*tert*-butyl 2,2',2''-(10-(2-((4-((6-((*tert*-butoxycarbonyl)amino)-2-(4-(4-iodophenyl)butanamido)hexanamido)methyl)benzyl)amino)-2-oxoethyl)-1,4,7,10-tetraazacyclododecane-1,4,7-triyl)(*S*)-triacetate (7, Scheme 2, step f)

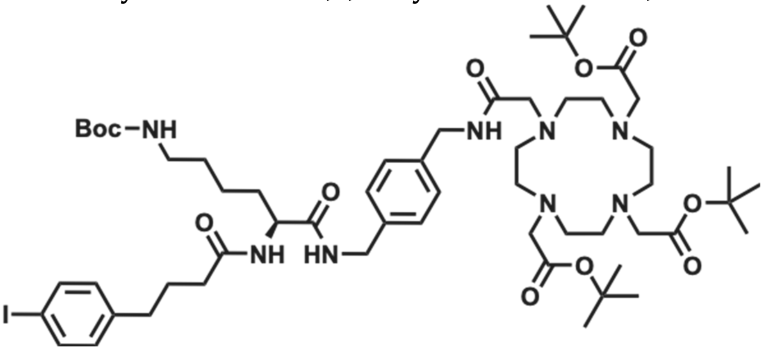

EDCi (7.3 mg, 47.2 µmol), HBTU (17.9 mg, 47.2 µmol), DOTA(tris-*t*Bu)ester (27 mg, 47.2 µmol) and DIPEA (13.7 µL, 78.6 µmol) were dissolved in DMF (0.5 mL) and stirred for 10 min. Then, compound **6** (20 mg, 31.5 µmol) in DMF (0.5 mL) was added and the resulting solution was stirred for one hour. The reaction mixture was concentrated under reduced pressure and purified by semi-preparative HPLC. Product was obtained as a white solid (9.85 mg, 8.27 µmol, 26%). HPLC (method A): purity >98%, t_R 17.2 min. ESI-MS: m/z calculated for $C_{57}H_{92}IN_8O_{11}^+$ was 1191.59, m/z found was 1213.40 $[M+Na]^+$, 1191.35 $[M+H]^+$, 546.18 $[M+2H]^{2+}$.

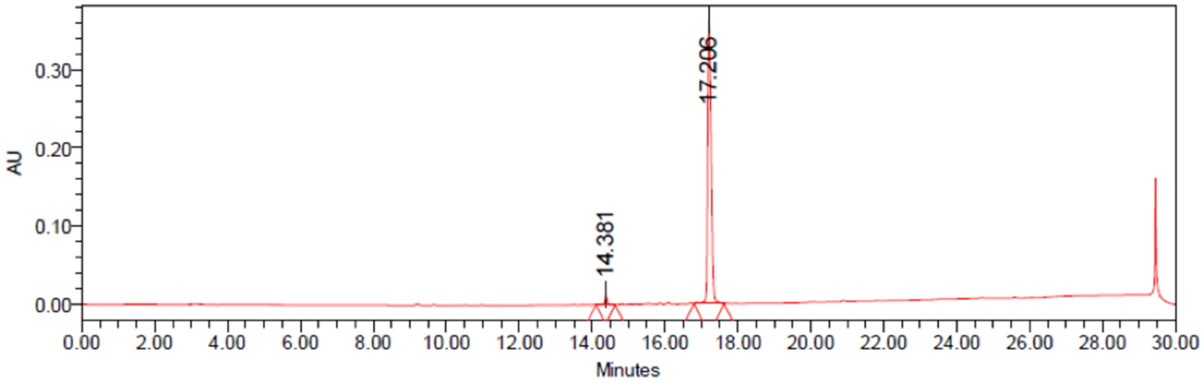

Figure S10: HPLC chromatogram of intermediate compound **7**.

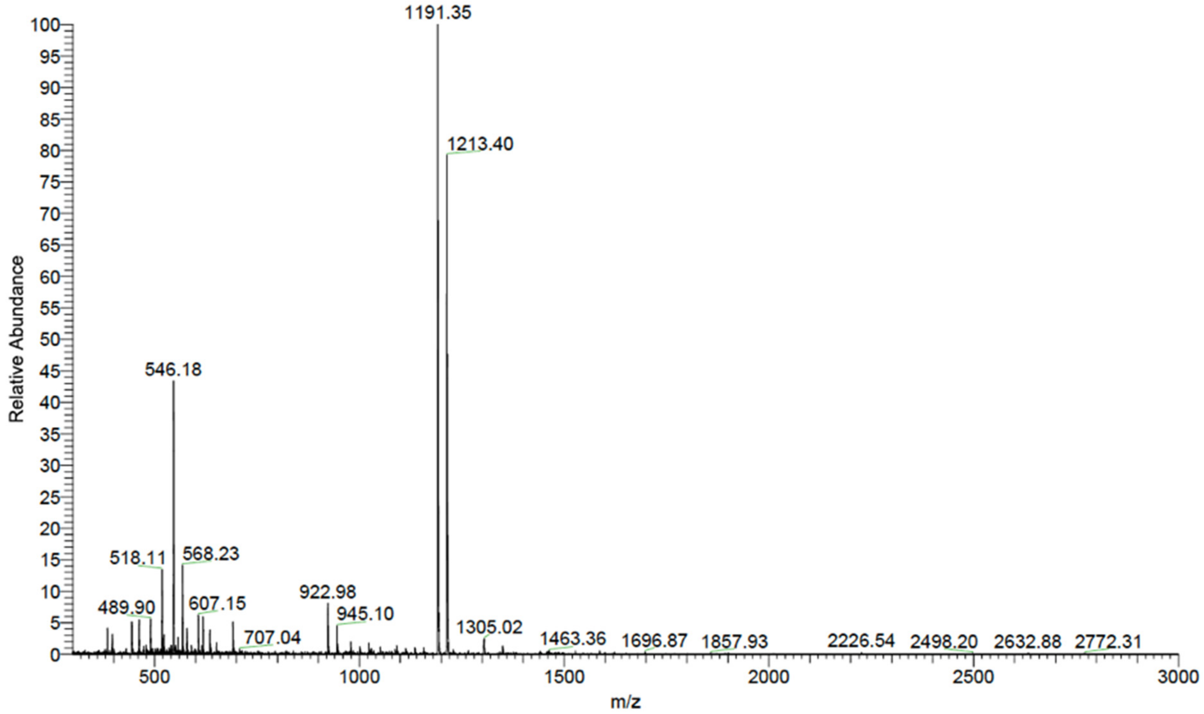

Figure S11: ESI-MS analysis of compound **7**.

1.7. (*S*)-2,2',2''-(10-(2-((4-((6-amino-2-(4-(4-iodophenyl)butanamido)hexanamido)methyl)benzyl)amino)-2-oxoethyl)-1,4,7,10-tetraazacyclododecane-1,4,7-triyl)triacetic acid (8, Scheme 2, step g)

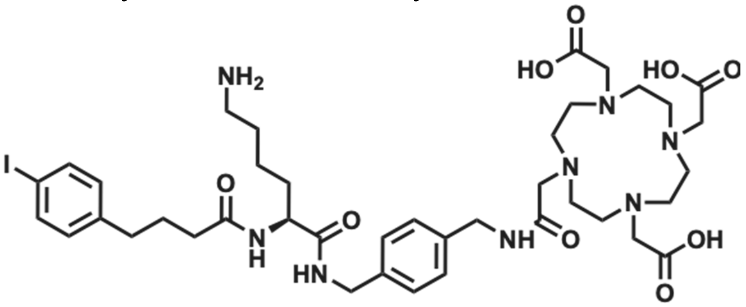

Compound **7** (1.92 mg, 2.08 μmol) was dissolved in a mixture of TFA:iP_3SiH:H_2O (95:2.5:2.5 v/v/v, 1.0 mL). After 3 hours, the crude mixture was diluted in Milli-Q water (10 mL) and the product was trapped on a C_{18} SepPac cartridge (Waters) eluted with Milli-Q water until neutral pH was reached. The intermediate was flushed from the cartridge with absolute ethanol and concentrated *in vacuo*. The residue was then purified by semi-preparative HPLC to obtain a white solid (1.96 mg, 1.89 μmol, 91%). HPLC (method A): purity >82%, t_R 11.9 min. ESI-MS: m/z calculated for $C_{40}H_{60}IN_8O_9^+$ was 923.35, m/z found was 945.17 $[M+Na]^+$, 923.12 $[M+H]^+$, 473.24 $[M+H+Na]^{2+}$, 462.11 $[M+2H]^{2+}$.

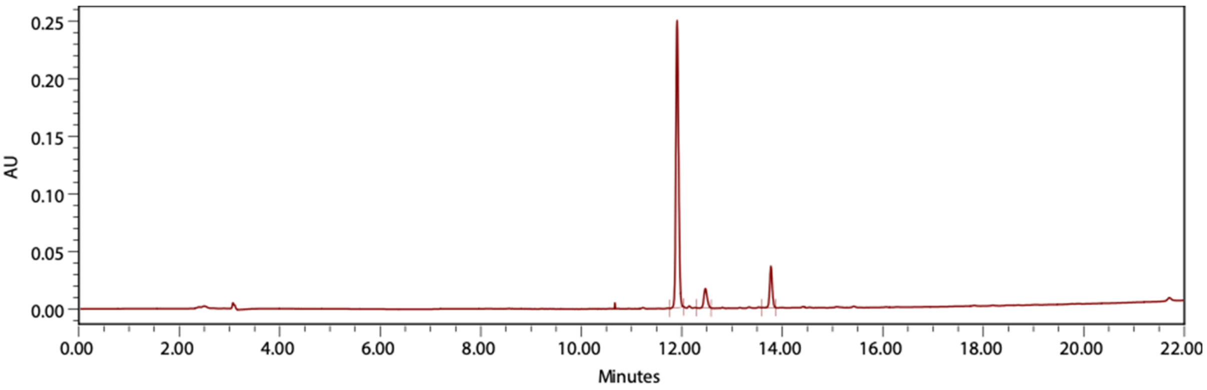

Figure S12: HPLC chromatogram of compound **8**.

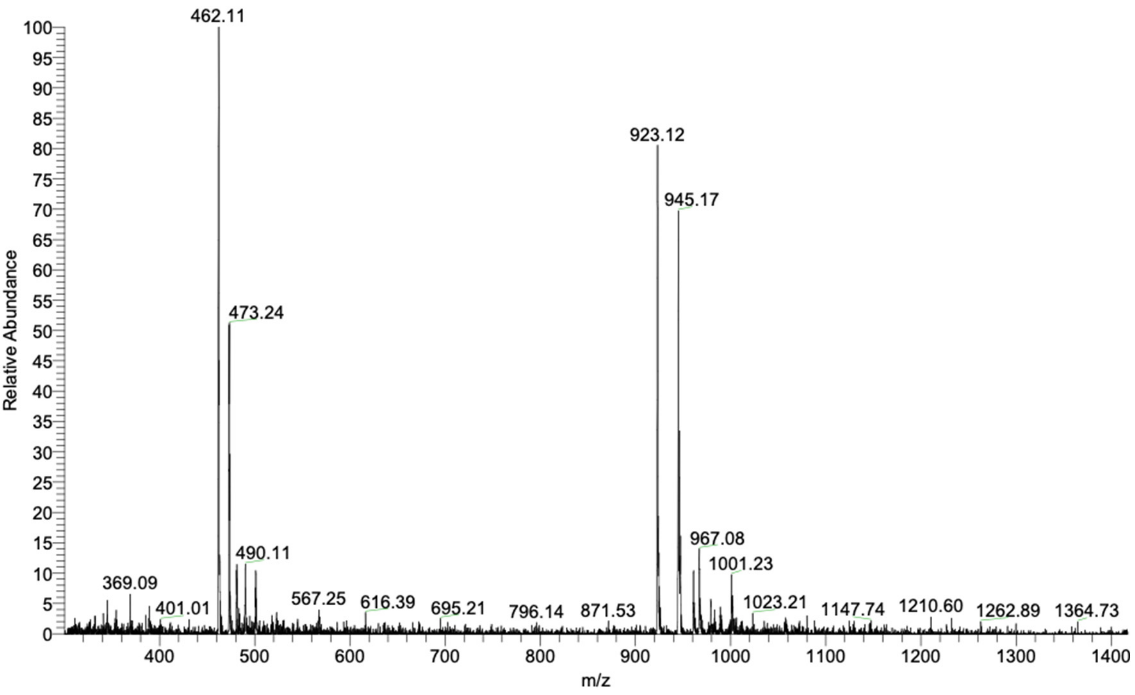

Figure S13: ESI-MS analysis of precursor **8**.

1.8. 4-(*p*-Iodophenyl)butyramide-L-Lys(IRDye800CW)-*p*-xylyleneamine-DOTA (3, step h)

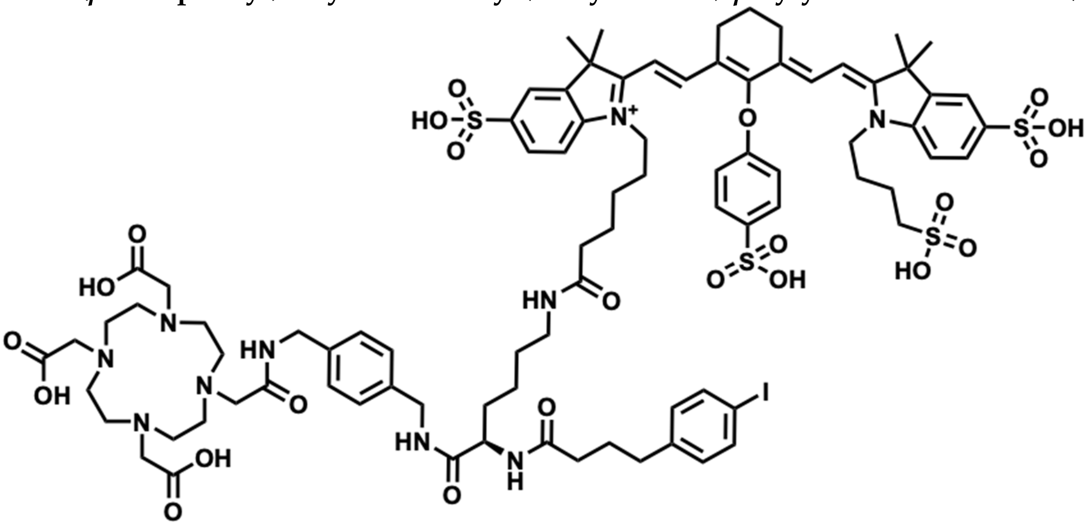

Deprotected amine **8** (1.66 mg, 1.7 µmol) dissolved in DMSO (250 µL) and DIPEA (1 µL, 5.7 µmol) was added to IRDye800CW-NHS (1.13 mg, 1.03 µmol). The resulting mixture was stirred at room temperature overnight and directly purified by semi-preparative HPLC (method C, t_R: 13.1 min) to yield **3** as a green solid (2.18 mg, 405 nmol, 39%). The precursor was titrated and stored in 10 nmol aliquots at -20 °C. HPLC (method D): purity >99%, t_R 12.3 min. ESI-MS: m/z calculated for $C_{86}H_{112}IN_{10}O_{23}S_4^+$ was 1907.58, m/z found was 954.27 $[M+2H]^{2+}$, 973.59 $[M+H+K]^{2+}$, 965.05 $[M+H+Na]^{2+}$.

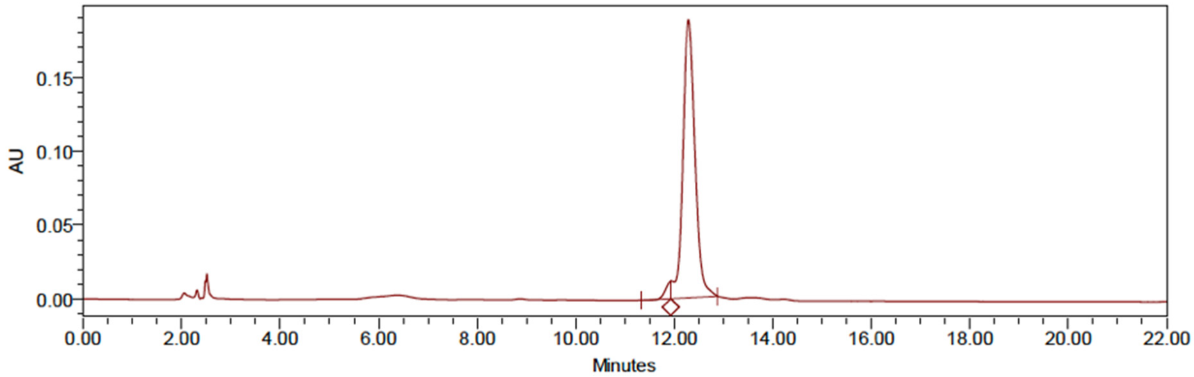

Figure S14: HPLC chromatogram of precursor **3**.

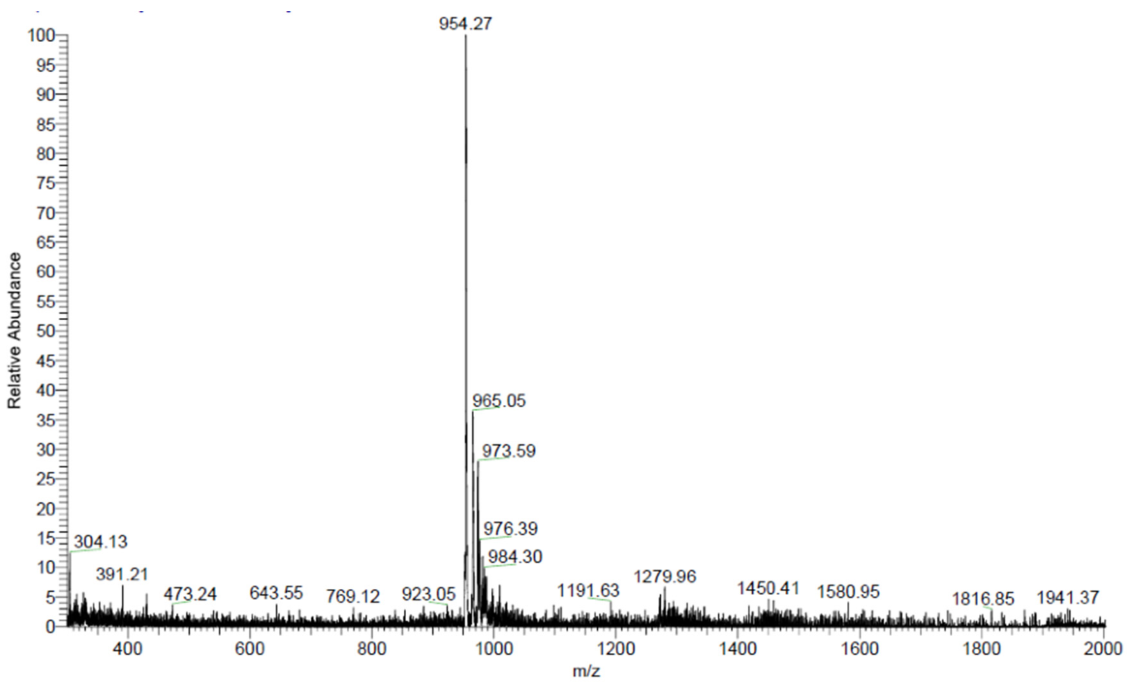

Figure S15: ESI-MS analysis of precursor **3**.

2. *Radiolabeling*

A conical vial was charged with precursor **1**, **2**, **3**, or **8** (30 µL, 10.0 nmol) and a gentisic acid/sodium ascorbate cocktail (both at 50 mM in 20 µL) was added to prevent radiolysis. The reaction mixture was brought to pH 4-4.5 with sodium acetate (10 µL, 2.5 M) and diluted with Milli-Q water to a final reaction volume of 140 µL. Then, [^{111}In]$InCl_3$ (100 MBq) was added and the sealed vial was heated to 90 °C for 15 min. The vial was cooled to room temperature for five minutes and DTPA (4 mM, 10 µL) was added[46]. Radiochemical purity (RCP) of [^{111}In]**2**/[^{111}In]**3** was >95% as determined by instant thin-layer chromatography (iTLC-SG paper, Aligant, eluent: 10% w/v NH_4Ac:MeOH 9:1). [^{111}In]DTPA eluted at the top of the paper while the indium-111 labeled NACAs remained at the baseline. RCP was confirmed by radio-HPLC (method D): >96%. Retention times were as followed: t_R([^{111}In]**2**): 12 min, t_R([^{111}In]**3**): 14 min and t_R([^{111}In]**8**): 12 min. Both tracers were tested for purity by HPLC (method D) after 24 hours in the reaction mixture.

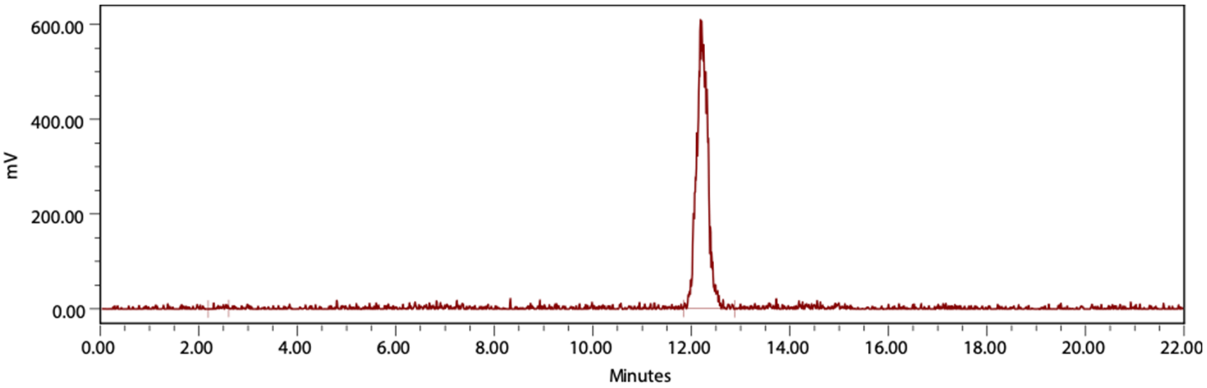

Figure S16: Radio-HPLC chromatogram of [^{111}In]**2**. Radiochemical purity >98%.

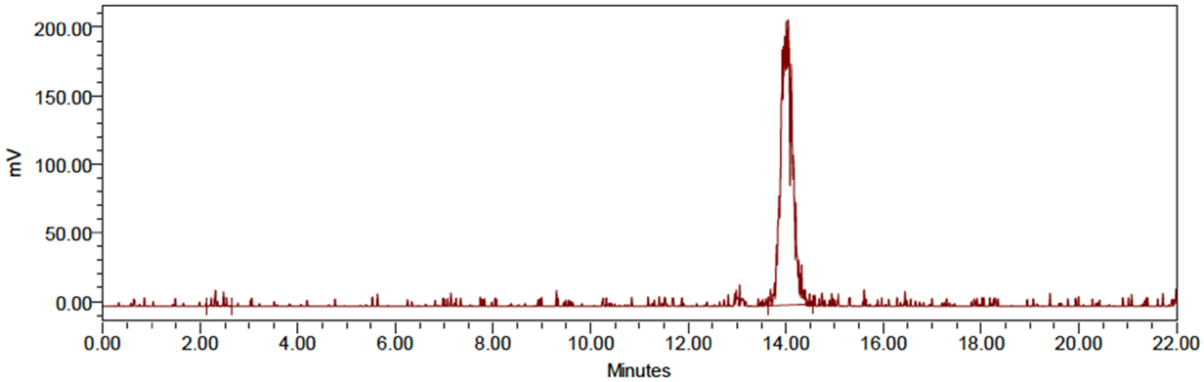

Figure S17: Radio-HPLC chromatogram of [^{111}In]**3**. Radiochemical purity of >96%.

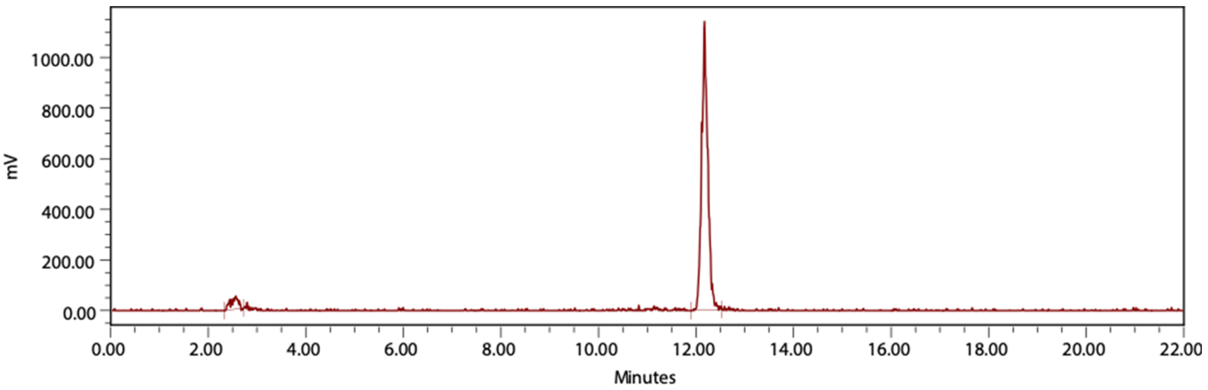

Figure S18: Radio-HPLC chromatogram of [^{111}In]**8**. Radiochemical purity of >93%.

3. In vivo *stability [^{111}In]**2** and [^{111}In]**3***

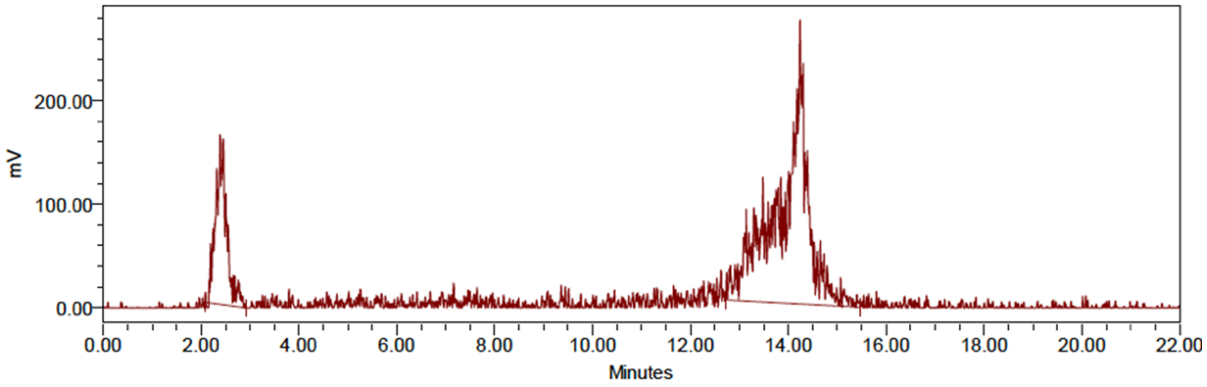

Figure S19: Radio-HPLC chromatogram of the urine from a mouse collected at one hour after injection of [^{111}In]**2**.

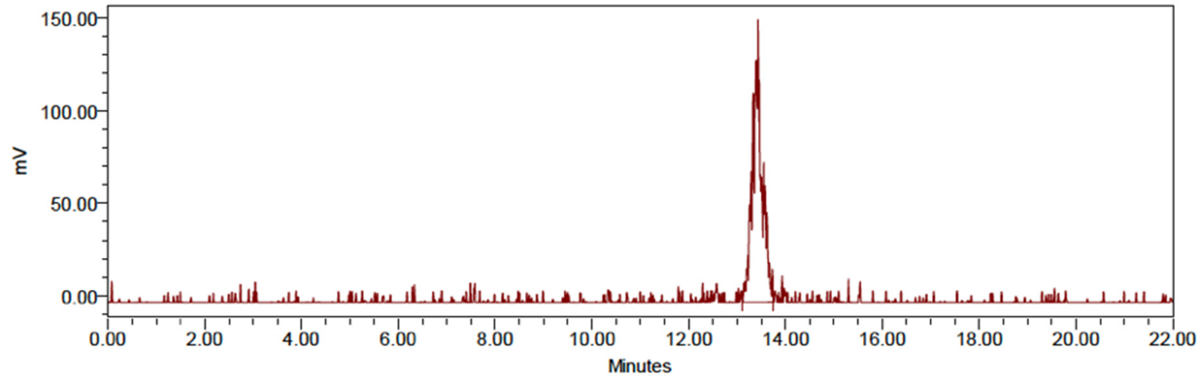

Figure S20: Radio-HPLC chromatogram of the urine from a mouse collected at one hour after injection of [^{111}In]**3**.

4. Four modality imaging experimental setup

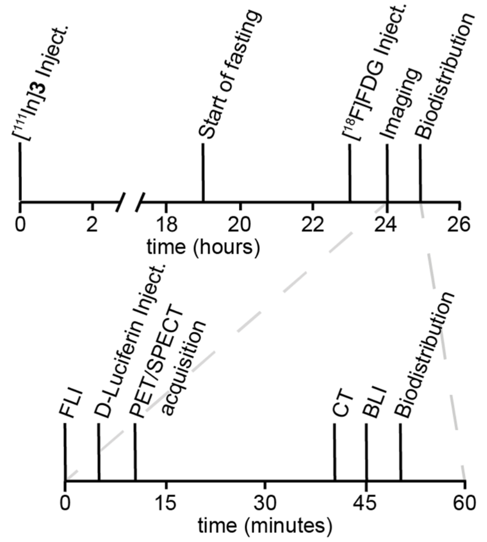

Figure S21: Time table for FLI, BLI, and PET/SPECT/CT tumor necrosis imaging procedure.

*5. Biodistribution data [^{111}In]**3***

Table S1. Biodistribution data from Figure 5. Data are presented as %ID/g tissue ± SEM (n = 4 for organs, n = 8 for tumors) after intravenous injection of [^{111}In]**3**.

	6 h p.i.	24 h p.i.	48 h p.i.	72 h p.i.
Blood	4.34±0.22	1.31±0.05	0.58±0.01	0.31±0.03
Skin	2.59±0.09	1.83±0.29	1.46±0.03	1.52±0.09
Pancreas	1.33±0.13	0.65±0.05	0.48±0.04	0.32±0.02
Liver	2.24±0.28	1.20±0.01	0.90±0.04	0.83±0.04
Spleen	1.87±0.18	1.45±0.03	1.19±0.07	1.10±0.18
Small intestine	0.93±0.07	0.53±0.05	0.33±0.01	0.20±0.02
Colon	1.60±0.13	0.70±0.07	0.37±0.02	0.23±0.02
Bone marrow	1.42±0.87	0.81%0.28	0.69%0.27	2.25±0.65
Kidneys	6.04±1.13	8.11%1.23	8.31±0.96	5.72±0.49
Lung	3.29±0.25	1.20±0.04	0.76±0.03	0.56±0.05
Heart	2.32±0.11	1.22±0.06	0.78±0.01	0.63±0.02
Muscle	1.31±0.20	0.46±0.03	0.36±0.03	0.26±0.03
Bone	1.63±0.16	0.93±0.13	0.74±0.04	0.51±0.05
Lymph nodes	3.91±0.97	2.42±0.18	3.30±0.62	2.39±0.25
Brain	0.14±0.03	0.07±0.00	0.04±0.00	0.03±0.00
4T1-Luc2 Tumors	3.94±0.19	3.27±0.25	2.62±0.23	2.25±0.64
Ratios				
Tumor to blood	0.91±0.05	2.50±0.20	4.54±0.45	7.36±0.93
Tumor to muscle	3.19±0.43	7.26±0.89	7.27±0.34	9.10±1.58
